# Protocol for a multi-site pilot and feasibility randomised controlled trial: Surgery versus PhysiothErapist-leD exercise for traumatic tears of the rotator cuff (the SPeEDy study)

**DOI:** 10.1186/s40814-020-00714-x

**Published:** 2021-01-07

**Authors:** Chris Littlewood, Julia Wade, Stephanie Butler-Walley, Martyn Lewis, David Beard, Amar Rangan, Gev Bhabra, Socrates Kalogrianitis, Cormac Kelly, Saurabh Mehta, Harvinder Pal Singh, Matthew Smith, Amol Tambe, James Tyler, Nadine E. Foster

**Affiliations:** 1 School of Primary, Community and Social Care, Keele University, Staffordshire, UK; 2 Department of Health Professions, Faculty of Health, Psychology & Social Care, Manchester Metropolitan University, Manchester, UK; 3 Population Health Sciences, Bristol Medical School, University of Bristol, Bristol, UK; 4 Keele Clinical Trials Unit, Keele University, Keele, UK; 5 Nuffield Department of Orthopaedics, Rheumatology and Musculoskeletal Sciences, University of Oxford, Oxford, UK; 6 Department of Health Sciences & Hull York Medical School, University of York, York, UK; 7 University Hospitals Coventry & Warwickshire, Coventry, UK; 8 Queen Elizabeth Hospital Birmingham, University Hospitals Birmingham NHS Foundation Trust, Birmingham, UK; 9 The Robert Jones and Agnes Hunt Orthopaedic Hospital NHS Foundation Trust, Oswestry, UK; 10 Royal Stoke University Hospital, University Hospitals of North Midlands NHS Trust, Stoke, UK; 11 Leicester Shoulder Unit, University Hospitals of Leicester NHS Trust, Leicester, UK; 12 The Liverpool Upper Limb Unit, The Royal Liverpool and Broadgreen University Hospitals NHS Trust, Liverpool, UK; 13 Derby Shoulder Unit, University Hospitals Derby & Burton NHS Foundation Trust, Derby, UK; 14 Airedale General Hospital, Airedale NHS Foundation Trust, Keighley, UK

**Keywords:** Surgery, Physiotherapy, Exercise, Rotator cuff tear, Shoulder, Randomised controlled trial

## Abstract

**Background:**

Clinically, a distinction is made between types of rotator cuff tear, traumatic and non-traumatic, and this sub-classification currently informs the treatment pathway. It is currently recommended that patients with traumatic rotator cuff tears are fast tracked for surgical opinion. However, there is uncertainty about the most clinically and cost-effective intervention for patients with traumatic rotator cuff tears and further research is required.

SPeEDy will assess the feasibility of a fully powered, multi-centre randomised controlled trial (RCT) to test the hypothesis that, compared to surgical repair (and usual post-operative rehabilitation), a programme of physiotherapist-led exercise is not clinically inferior, but is more cost-effective for patients with traumatic rotator cuff tears.

**Methods:**

SPeEDy is a two-arm, multi-centre pilot and feasibility RCT with integrated Quintet Recruitment Intervention (QRI) and further qualitative investigation of patient experience. A total of 76 patients with traumatic rotator cuff tears will be recruited from approximately eight UK NHS hospitals and randomly allocated to either surgical repair and usual post-operative rehabilitation or a programme of physiotherapist-led exercise. The QRI is a mixed-methods approach that includes data collection and analysis of screening logs, audio recordings of recruitment consultations, interviews with patients and clinicians involved in recruitment, and review of study documentation as a basis for developing action plans to address identified difficulties whilst recruitment to the RCT is underway. A further sample of patient participants will be purposively sampled from both intervention groups and interviewed to explore reasons for initial participation, treatment acceptability, reasons for non-completion of treatment, where relevant, and any reasons for treatment crossover.

**Discussion:**

Research to date suggests that there is uncertainty regarding the most clinically and cost-effective interventions for patients with traumatic rotator cuff tears. There is a clear need for a high-quality, fully powered, RCT to better inform clinical practice. Prior to this, we first need to undertake a pilot and feasibility RCT to address current uncertainties about recruitment, retention and number of and reasons for treatment crossover.

**Trial registration:**

ClinicalTrials.gov (NCT04027205) – Registered on 19 July 2019. Available via

## Introduction

### Background and rationale

Shoulder pain presents a significant personal, social and economic burden and impacts on work, ability to undertake leisure and household tasks and causes disturbed sleep [1]. Tears of the rotator cuff are regarded as a significant cause of shoulder pain and rates of surgery to repair the torn rotator cuff have risen approximately 200% over recent years across Europe and the USA [2–5]. In the UK NHS, 8838 surgical repairs of the rotator cuff were undertaken in 2018/2019 with approximately one-third undertaken for traumatic tears [6]. Depending on complexity, the cost of surgical repair ranges from £3676 to £6419 [7] meaning that direct NHS treatment costs alone range from £26.6 to £51.4 million annually, and £10.8 to £18.9 million specifically for traumatic rotator cuff tears.

Different treatment pathways are proposed in the current British Elbow & Shoulder Society and British Orthopaedic Association guidelines [8] for patients presenting with non-traumatic as opposed to traumatic rotator cuff tears. These guidelines recommend that patients with traumatic rotator cuff tears are fast tracked for surgical opinion. Three randomised controlled trials (RCTs) (*n* = 252) comparing surgery to non-surgical treatment for rotator cuff tears have been undertaken and synthesised in a systematic review (SR) [9]. The review concluded that, although the evidence is limited in terms of quality, current research suggests no difference in clinical outcomes at 1 year between surgery and non-surgical treatment. However, of the 252 patients included in the SR, only 40 (16%) were diagnosed with traumatic tears of the rotator cuff (24 randomised to surgery; 16 to physiotherapist-led exercise). Since the publication of this SR, one further RCT (*n* = 58) focusing specifically on traumatic rotator cuff tears has been published [10]. This RCT compared surgical repair with a programme of physiotherapist-led exercise for acute traumatic rotator cuff tears located mainly within supraspinatus [10]. At 12 months, there was no significant between-group difference in terms of shoulder pain and function. Re-tear was reported in 6.5% of participants undergoing repair and tear size progression greater than 5 mm in 29.2% of participants undergoing the programme of physiotherapist-led exercise [10]. Hence, there is uncertainty about optimal interventions for traumatic rotator cuff tears based on evidence from a limited number of patients and from RCTs with relatively short-term follow-up.

Despite this uncertainty from RCTs, other reasons for current guidelines recommending fast track for surgical opinion for people with traumatic rotator cuff tears include a concern that a delayed surgical repair is more technically challenging and that delay risks poorer clinical outcomes. Several non-randomised studies have evaluated the impact of time to surgery on clinical outcomes. Findings vary considerably with some recommending surgery within 4 months of symptom onset [11], some 6 months [12] and some 24 months [13], yet others conclude that time to surgery is not a critical factor [14]. Thus, there is considerable uncertainty and given that asymptomatic rotator cuff tears are very common, it is also difficult to attribute tears of the rotator cuff to recent trauma with confidence [15]. Imaging for shoulder pain following trauma may in fact be identifying an existing asymptomatic rotator cuff tear. Another reason proposed in support of current guidelines recommending fast track for surgical opinion relates to progression, i.e. the increasing size of a rotator cuff tear if not operated on [16]. It has been reported that 42 to 47% of symptomatic tears increase in size up to 100 months follow-up [16, 17] with the greatest rate of increase in those with full-thickness tears observed in 82% versus 26% of those with partial-thickness tears [17]. Hence, most full thickness and some partial-thickness rotator cuff tears do increase in size over time, but it is apparent that some do not and, importantly, surgery is not guaranteed to prevent progression and these increases in the size of tear are not consistently associated with poorer clinical outcome in terms of pain and function [17, 18]. To further highlight uncertainty in the management of rotator cuff tears, the UKUFF trial [1] reported a 40% re-tear or failed-healing rate following surgical repair of non-traumatic rotator cuff tears but the outcomes for these patients were not clinically significantly different from those patients who did not re-tear their rotator cuff. Similar findings have also been reported in a systematic review and meta-analysis of 14 studies (*n* = 861) [19]. This suggests that the tear might not be the only source of symptoms and surgical repair, in some circumstances, might be unwarranted. In situations where both the source of symptoms and the mechanism of action of surgery are questioned, a physiotherapist-led exercise might be a credible treatment option just as it currently is for patients with non-traumatic tears [20].

The above uncertainty and lack of evidence to inform decision-making provides the justification for the current research study.

### Objectives

The aim of the SPeEDy study is to determine if it is feasible to conduct a future, substantive, multi-site RCT to test the hypothesis that physiotherapist-led exercise is not inferior to surgical repair of the rotator cuff in terms of clinical outcomes but is more cost-effective.

The following objectives are to:

1. Determine feasibility of recruiting patients
2. Determine feasibility of retaining participants, including response rates to questionnaires
3. Determine zone of clinical equipoise, including exploration of what patient characteristics (age, size, and location of rotator cuff tear etc.) influence whether clinicians are prepared to randomise patients or not
4. Determine proportion and reasons for treatment crossover
5. Estimate the number of potential and willing sites for the future main RCT
6. Identify barriers and facilitators to recruitment and retention
7. Determine participant satisfaction with the interventions
8. Determine the number and nature of adverse events

### Trial design

**Fig. 1:**
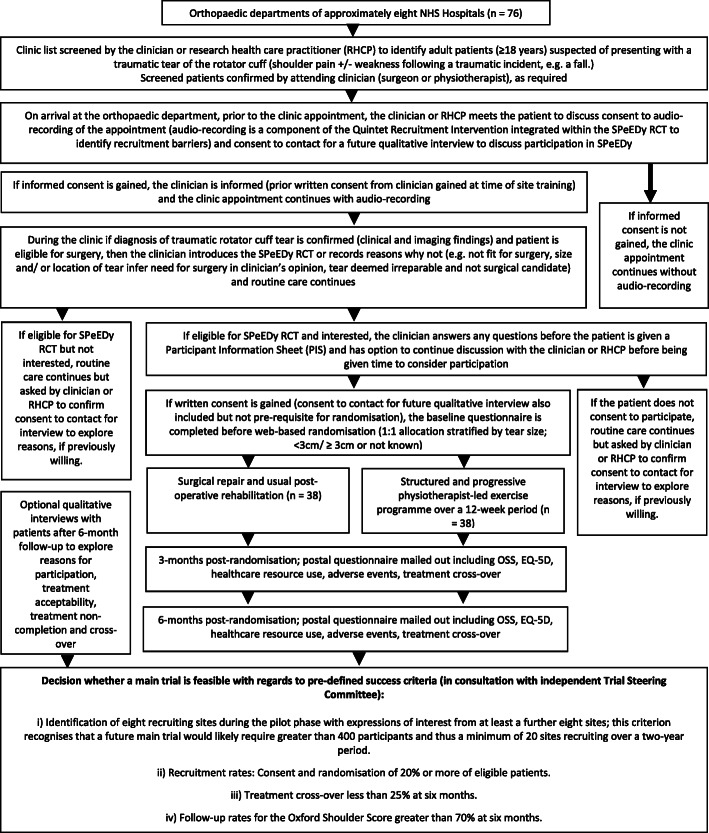
Study flow chart

**Fig. 2:**
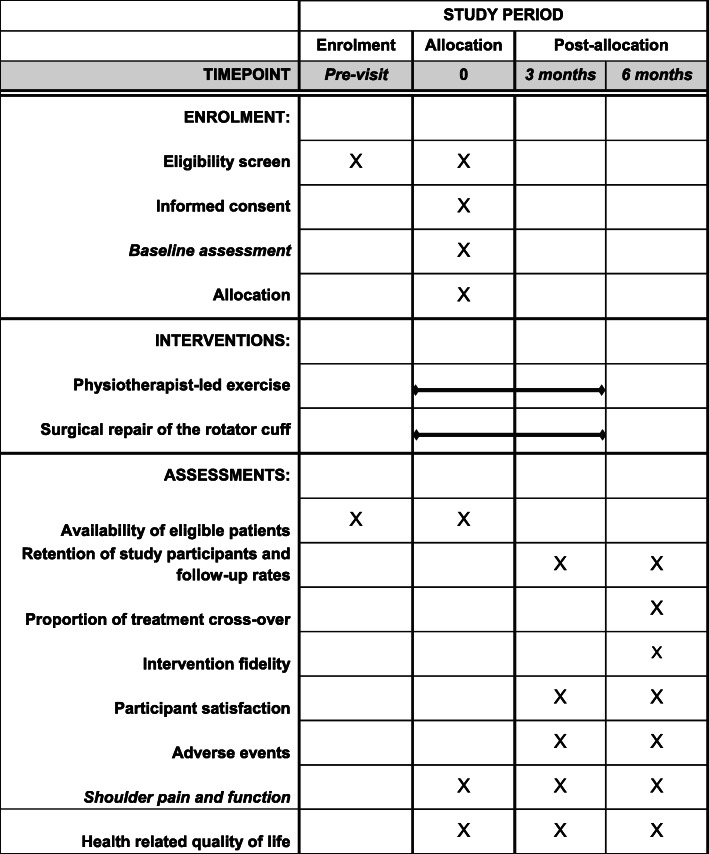
Schedule of enrolment, interventions and assessments (SPIRIT Statement) [21]

The SPeEDy study is a two-arm, multi-centre pilot and feasibility RCT (1:1 allocation ratio) with integrated Quintet Recruitment Intervention (QRI) and further qualitative investigation of patient experience (Figs. 1 and 2).

## Methods: Participants, interventions and outcomes

### Study setting

Patients will be recruited from orthopaedic departments of the participating UK NHS hospitals. The study interventions will be delivered through their associated orthopaedic and physiotherapy services.

### Eligibility criteria

#### Inclusion criteria

1. Adult patients (≥ 18 years)
2. Diagnosed with a symptomatic tear of the rotator cuff following a traumatic incident thought to be of sufficient force to induce a tear
3. Rotator cuff tear confirmed by diagnostic ultrasound or MRI scan undertaken as part of routine diagnostic workup
4. Eligible for rotator cuff repair surgery or a programme of physiotherapist-led exercise as determined by the attending clinician (surgeon or physiotherapist, where appropriate)
5. Able to return to the participating NHS hospital or associated orthopaedic and physiotherapy services (where physiotherapists have been trained in trial interventions) for post-operative rehabilitation or the programme of physiotherapist-led exercise
6. Able to understand English

#### Exclusion criteria

1. Not eligible for rotator cuff repair surgery or a programme of physiotherapist-led exercise as determined by the attending clinician (surgeon or physiotherapist, where appropriate)
2. Patients who are unable to give full informed consent

### Who will take informed consent?

A local clinician or local research healthcare practitioner, who will have received appropriate training and is authorised on the trial delegation log, will support the process of informed consent. Interested and eligible patients will be required to provide written informed consent before participating (see reference below to optional Quintet Recruitment Intervention (QRI) and optional 6-month qualitative interviews). Written informed consent for the QRI will be sought at the time of attendance at the orthopaedic clinic. Consent for the RCT will be sought at the time of attendance at the orthopaedic clinic or other convenient time prior to the surgery if the patient requests more time to consider participation.

The RCT consent form indicates if the participant would like to be contacted for the purpose of the 6-month qualitative interviews. If the participant indicates yes, a researcher will contact the participant and if they agree to take part, a subsequent consent form will be signed.

### Interventions

#### Explanation for the choice of comparators

Fast track for surgical opinion and surgical repair of the traumatic rotator cuff tear is the current standard of care and this will be compared with a programme of physiotherapist-led exercise, should this prove feasible during this pilot phase, testing the hypothesis that the latter is not clinically inferior to the standard of care but is more cost-effective.

#### Intervention description

##### Surgical repair plus usual post-operative rehabilitation

Surgical repair will be guided by the size and location of the tear and also surgeon preference, the details of which will be recorded on a specific case report form and reported accordingly. Similarly, the content of post-operative rehabilitation is variable across the UK but typically begins with a period of immobilisation using an arm sling for up to 6 weeks. After this period, rehabilitation progresses gradually with the aim of restoring movement, strength and function [22].

##### Structured and progressive physiotherapist-led exercise programme

The exercise programme is based on the principle of self-dosing with the aim of restoring functional capacity to a level acceptable to the individual participant. Exercise prescription is based on establishing the current functional capacity of the patient in relation to the most challenging shoulder movements and is supported over approximately six contact sessions with a physiotherapist across a 12-week period. The development process and resultant programme of physiotherapist-led exercise used in the SPeEDy study has been reported elsewhere in full [23].

#### Criteria for discontinuing or modifying allocated interventions

There are no special criteria for discontinuing or modifying allocated interventions.

Participants may choose to withdraw from the allocated intervention for any reason and continue a plan of treatment determined in consultation with their attending clinician.

#### Strategies to improve adherence to interventions

Adherence to the physiotherapist-led exercise programme will be recorded in an exercise diary provided to the patient and monitored by the physiotherapist. No further strategies beyond usual encouragement will be used.

#### Relevant concomitant care permitted or prohibited during the trial

No special provisions.

#### Provisions for post-trial care

None beyond standard care within the NHS.

#### Outcomes

##### Feasibility outcomes

1. Numbers of patients screened, number eligible, number approached, number consenting, number randomised and number accepting allocation and reasons for patients not being eligible, approached or declining participation
2. Numbers of participants receiving and completing the allocated intervention
3. Follow-up response rates to questionnaires at 3 and 6 months post-randomisation (including Oxford Shoulder Score and EQ-5D-5L)
4. Determine reasons for not approaching potentially eligible patients and how this varies between clinicians
5. Numbers of participants receiving intervention (surgery or PT-led exercise) other than that which was allocated to determine proportion of participants who crossover
6. Numbers of sites agreeing to participate and numbers of additional sites who are interested in participating in the main trial
7. Participant satisfaction with the interventions on a five-point ordinal scale; very satisfied/satisfied/neutral/dissatisfied/very dissatisfied
8. Barriers and facilitators to recruitment, retention and treatment crossover (qualitative data; audio recording and individual interviews)

##### Clinical outcomes

1. Pain and disability assessed using the Oxford Shoulder Score (OSS) at baseline, 3 and 6 months post-randomisation by post with telephone call for minimum data collection if no response to reminder postal questionnaire
2. Health-related quality of life assessed using the EQ-5D-5L at baseline, 3 and 6 months post-randomisation by post with telephone call for minimum data collection if no response to reminder postal questionnaire
3. Days lost from work due to the shoulder problem at 3 and 6 months post-randomisation via postal questionnaire
4. Time taken to return to driving, if applicable, via questionnaire at 3 and 6 months post-randomisation via postal questionnaire
5. Number and type of adverse events for up to 6 months post-randomisation via patient self-report questionnaire or telephone Minimal Data Collection (MDC) at 3 and 6 months and via surgeon, physiotherapist or GP report

Self-report data relating to further healthcare resource use, including NHS- and private-borne service, and medication costs will also be collected.

#### Participant timeline

See s. 1 and 2.

#### Sample size

Randomising 76 participants will enable the 1-sided lower 90% confidence limit for the follow-up rate to be estimated within about 6% of the anticipated 80% level at 6 months. Further, a sample size of about 76, allowing for dropout, will be sufficient to allow precise calculation of estimates of standard deviation around the OSS for the main RCT [24].

Additionally, with reference to treatment crossover of less than 25% (principal concern relates to those randomised to the physiotherapist-led exercise programme crossing over to surgery), randomising 38 participants to the physiotherapist-led exercise programme enables precise 1-sided upper confidence limits (e.g. around 8% above the desired upper level of around 15% for a 90% upper confidence limit).

#### Recruitment

Potential participants will be identified when the diagnosis of symptomatic traumatic rotator cuff tear is made (sudden onset of shoulder pain and weakness following a traumatic incident with a tear confirmed by ultrasound or MRI) and surgery is considered a treatment option. The patient pathway is variable between sites but, typically, this diagnosis and identification of the two possible management options will be made by an orthopaedic surgeon, or physiotherapist, who will subsequently introduce the RCT to the patient. If the patient expresses interest, further discussion will ensue between the patient and the clinician or research healthcare practitioner (depending on availability) who will provide them with a study information pack, by hand or in the post, and follow this up with a discussion, face-to-face or over the telephone.

### Assignment of interventions: Allocation

#### Sequence generation

Participants will be allocated on a 1:1 ratio, stratified by tear size (large tear ≥ 3 cm/small to medium sized tear < 3 cm/or not known), using mixed-block randomisation.

#### Concealment mechanism

Randomisation will be undertaken remotely using web-based randomisation to ensure allocation concealment provided by Derby Clinical Trials Support Unit.

#### Implementation

The participant will be informed of the allocation by the clinician or research healthcare practitioner, depending on availability, and will then access the interventions as per usual treatment pathways, including being placed on a waiting list for surgery or physiotherapy.

### Assignment of interventions: Blinding

#### Who will be blinded

No measures to blind participants, clinicians, research team or oversight committees will be implemented.

### Data collection and management

#### Plans for assessment and collection of outcomes

Local sites will complete screening logs and participants will complete the baseline questionnaire, including demographic data, the Oxford Shoulder Score (OSS) and EQ-5D-5L. The OSS is a 12-item shoulder-specific self-report measure of shoulder pain and function primarily for the assessment of outcome of shoulder surgery in RCTs [25]. The OSS is reliable, valid, responsive and acceptable to patients [1, 25, 26]. The items refer to the past 4 weeks with five ordinal response options scored from 0 to four, with four representing the best outcome. When the 12 items are summed, this produces an overall score ranging from 0 to 48, with 48 being the best outcome.

The EQ-5D-5L is a generic measure of health-related quality of life that provides a single index value for health status that can be used for the purpose of clinical and health economic evaluation [27]. The EQ-5D-5L consists of questions relating to five health domains (mobility, self-care, usual activities, pain/discomfort, anxiety/depression), and respondents rate their degree of impairment using five response levels (no problems, slight problems, moderate problems, severe problems and extreme problems) [27]. EQ-5D is the National Institute for Health and Care Excellence’s (NICE) preferred measure of health-related quality of life in adults.

Follow-up at 3 and 6 months post-randomisation will be via postal questionnaire.

#### Plans to promote participant retention and complete follow-up

Follow-up processes include reminders and MDC via telephone if questionnaires are not returned within 2 and 4 weeks respectively.

#### Data management

All participants are given an individual study number that will be used on all case report forms for that participant. Case report forms will be processed and stored securely.

#### Confidentiality

All collected information will be kept strictly confidential and will be stored in accordance with the General Data Protection Regulations (GDPR) and retained in accordance with the latest Directive on Good Clinical Practice (GCP) and local policy.

### Statistical methods

#### Statistical methods for primary and secondary outcomes

As this is a pilot and feasibility RCT, the main analysis will focus on process outcomes, including consent rate, retention rate and follow-up rates. Means and confidence intervals of the OSS will be calculated in order to inform the sample size calculation for the main RCT. Analysis will be undertaken using the IBM SPSS statistics package (https://www.ibm.com/uk-en/products/spss-statistics). A detailed data analysis plan will be agreed with the independent trial steering committee (TSC) before any analysis is undertaken.

The following success criteria will be used to decide on whether to proceed to developing a main RCT:

i) Identification of eight recruiting sites during the pilot phase with expressions of interest from at least a further eight sites
ii) Recruitment rates: Consent and randomisation of 20% or more of eligible patients
iii) Treatment crossover less than 25%, of those randomised, at 6 months in each of the two treatment groups
iv) Follow-up rates for the OSS greater than 70% at 6 months

#### Interim analyses

No interim analyses are planned.

#### Methods for additional analyses (e.g. subgroup analyses)

No additional analyses are planned.

#### Methods in analysis to handle protocol non-adherence and any statistical methods to handle missing data

The primary analysis will be intention to treat. However, an additional per-protocol analysis will be undertaken to account for any participants who did not receive the allocated intervention within the study period, with no account taken of protocol non-adherence beyond reporting of treatment crossover.

### The Quintet recruitment intervention

RCTs can be challenging to recruit to, with clear obstacles including a lack of time and strong patient preferences, but also hidden challenges including tensions for clinicians and challenges around assuming a position of equipoise [28, 29]. However, without systematic evaluation, hidden challenges remain unknown and opportunities to change RCT processes, for example, study training and documentation, are lost to the potential detriment to the RCT. In recognition of this, the QRI will be integrated within the SPeEDy RCT to understand the challenges associated with recruitment and develop action plans to address these challenges rapidly whilst recruitment is underway.

The QRI is a mixed-methods approach that includes data collection and analysis of screening and eligibility logs, audio recordings of recruitment consultations (i.e. consultations during which recruitment to the RCT is discussed), individual interviews with patients and clinicians involved in recruitment, and review of study documentation as a basis for developing action plans to address identified difficulties whilst recruitment to the RCT is underway [30, 31]. It has been shown to facilitate recruitment to the most challenging RCTs, including orthopaedic RCTs [29, 32]. A targeted QRI, investigating recruiter information provision, patient responses to this information and recruitment performance across recruiters/sites, will be integrated within the SPeEDy RCT over the recruitment period to enable barriers to recruitment to be identified and any changes to the recruitment process to be made and re-evaluated.

In line with the SEAR (Screening, Eligibility, Approached, Randomised) framework [33], screening and eligibility logs will detail all patients screened (by the research healthcare practitioner (RHCP) and attending clinician) to identify how many are eligible, approached, accepting randomisation and the allocated intervention, alongside reasons not eligible, approached or accepting randomisation. These will be completed at the clinical site.

Clinic and recruitment appointments involving discussion of the SPeEDy RCT will be audio recorded and then subjected to targeted data extraction and transcription, focusing analysis on discussion of issues relevant to recruitment to the SPeEDy RCT, to investigate how recruiters present the study to patients and how patients respond.

In-depth, semi-structured interviews will be conducted, guided by topic guides with a purposive sample of patients and clinicians involved in identifying patients and RHCPs (responsible for explaining the trial and obtaining informed consent) from selected sites. Selection of sites will be determined by willingness to participate in the QRI and determined by relative recruitment performance, i.e. high and low-recruiting sites.

Given the responsive and iterative nature of the QRI, it is not realistic to pre-specify a required number of participants. But, we will aim to sample data from four out of the eight-hospital sites including six to 12 clinicians and 12‑20 patients or until saturation is reached. Data collection will be limited to the pre-specified maximum 9-month period. However, data collection will cease if no new themes emerge from the ongoing analysis prior to this time point.

Data from the screening and eligibility logs will be analysed descriptively to identify differences between sites and points in the recruitment pathway where patients at particular sites are ‘lost’ to recruitment. Findings from this analysis will guide sampling for audio recording of recruitment discussions and interviews.

Audio recordings of clinic and recruitment appointments will be analysed using thematic, content and targeted conversation analysis techniques with the aim of understanding the challenges to recruitment [34]. In tandem with this, data from individual interviews will be analysed using constant comparison techniques which involves detailed coding followed by comparison of emerging themes and codes within transcripts and across the dataset looking for shared or disparate views. Findings will be presented anonymously.

Study documentation, including PIS and consent forms and Trial Management Group (TMG)/Trial Steering Committee (TSC) meeting minutes, will be reviewed with reference to the findings from the interviews and recruitment appointments. Data from screening logs, audio recordings, interviews and study documentation will be triangulated to identify where findings are confirmed across data sources.

Upon completion of this analysis, an action plan will be generated to address factors that appear to be hindering recruitment. Previous reports of the outcome of the QRI have identified generic and study-specific barriers to recruitment which can be addressed through bespoke action plans [29]. Also in some instances, the QRI has provided clear reasons why recruitment to the RCT might not be achievable [29]. Once the action plan is agreed, where relevant, study documentation will be amended and further training offered to recruiters across all of the hospital sites. Post-implementation of the plan, a further evaluation of the recruitment process will be undertaken using feasibility outcome data described in this protocol.

### Patient experience of trial participation

Following the 6-month follow-up point, a further sample of patient participants will be purposively sampled from both intervention groups and interviewed to explore reasons for initial participation, treatment acceptability, reasons for non-completion of treatment, where relevant, and any reasons for treatment crossover.

Patients will be purposefully sampled with respect to allocated group, change in pain and disability status according to the OSS, whether the allocated treatment was completed or not and in relation to treatment crossover. Patients will be able to decline participation in the interviews yet still be involved in the RCT.

The interviews will be based not only on semi-structured topic guides developed in relation to the pre-specified aims but also with our patient and public involvement group (approximately 30-min telephone interview). It is expected that approximately 20 patients will be sufficient to attain rich data. Telephone interviews will be conducted, audio recorded and transcribed ad verbatim.

As with the QRI qualitative data analysis, interviews will be analysed thematically.

#### Plans to give access to the full protocol, participant level-data and statistical code

The full protocol is available via clinicaltrials.gov (https://clinicaltrials.gov/ct2/show/NCT04027205). In the first instance, further requests for data can be made via the corresponding author.

### Oversight and monitoring

#### Composition of the coordinating centre and trial steering committee

The chief investigator (CL) is responsible for the conduct of the study and will be supported by the TMG comprising members of the research team.

An independent TSC will oversee the study and includes an independent chair and four further independent members comprising a statistician, lay and clinical representatives. The CI, senior statistician and Trial Manager will attend the TSC meetings and present and report progress. TSC meetings will be scheduled approximately annually.

#### Composition of the data monitoring committee, its role and reporting structure

Since this is a pilot RCT with no planned interim statistical analysis, a Data Monitoring Committee has not been formed and the TSC has agreed to take responsibility for reviewing the safety of the trial including advice regarding progression to a main RCT with reference to the pre-specified progression criteria.

#### Adverse event reporting and harms

Number and type of adverse events for up to 6 months post-randomisation will be collected via patient self-report questionnaire or telephone MDC at 3 and 6 months and via surgeon, physiotherapist or GP report.

#### Frequency and plans for auditing trial conduct

Independent TSC meetings will be scheduled approximately annually.

#### Plans for communicating important protocol amendments to relevant parties (e.g. trial participants, ethical committees)

Funders, sponsors and NHS Research & Development Offices will be notified routinely and appropriate approvals gained and communicated as required by them and by the study sponsor.

#### Dissemination plans

The results of the SPeEDy study will be published in peer-reviewed journals, presented at relevant conferences and disseminated to clinicians and patients, as appropriate.

## Discussion

Despite a dearth of research evidence and uncertainty regarding the most clinical and cost-effective treatment interventions for patients with traumatic rotator cuff tears, current national guidelines suggest that people with suspected traumatic tears should seek urgent surgical opinion [8]. It remains unclear whether such an approach should be recommended in preference to, for example, non-surgical assessment and management. Recognising current uncertainties, SPeEDy has been developed as a pilot and feasibility RCT with integrated QRI and further qualitative interviews to investigate the barriers, and facilitators, to recruitment, intervention delivery and fidelity, and clinical equipoise from the perspectives of patients and clinicians. We expect recruitment to be challenging but there is a clear need for a high-quality, fully powered, RCT to compare surgery and usual post-operative rehabilitation versus a programme of physiotherapist-led exercise for a defined group of patients presenting with traumatic rotator cuff tears. This pilot and feasibility RCT will determine the feasibility of a fully powered RCT, including whether it is possible to recruit patients and retain participants in their allocated group.

### Study status

The SPeEDy study (protocol version 1.0 dated 05 August 2019) opened to recruitment on 3 March 2020, and was scheduled to complete recruitment by 31 January 2021. However, recruitment was paused due to COVID-19 on 16 March 2020, re-opened on 3 August 2020, but then paused again on 4 October 2020, to enable transfer of the study sponsor.

## Data Availability

Data required to support the protocol will be supplied on request to the corresponding author.
